# Nonpharmaceutical Interventions for Pandemic Influenza, National and Community Measures

**DOI:** 10.3201/eid1201.051371

**Published:** 2006-01

**Authors:** World Health Organization Writing Group

**Keywords:** influenza, World Health Organization, quarantine

## Abstract

The World Health Organization's recommended pandemic influenza interventions, based on limited data, vary by transmission pattern, pandemic phase, and illness severity and extent. In the pandemic alert period, recommendations include isolation of patients and quarantine of contacts, accompanied by antiviral therapy. During the pandemic period, the focus shifts to delaying spread and reducing effects through population-based measures. Ill persons should remain home when they first become symptomatic, but forced isolation and quarantine are ineffective and impractical. If the pandemic is severe, social distancing measures such as school closures should be considered. Nonessential domestic travel to affected areas should be deferred. Hand and respiratory hygiene should be routine; mask use should be based on setting and risk, and contaminated household surfaces should be disinfected. Additional research and field assessments during pandemics are essential to update recommendations. Legal authority and procedures for implementing interventions should be understood in advance and should respect cultural differences and human rights.

## Abstract 2

Recommended interventions vary by transmission pattern, pandemic phase, and disease severity.

This article is the second of a 2-part series that summarizes the scientific basis for nonpharmaceutical public health interventions recommended by the World Health Organization (WHO) to contain or reduce transmission of pandemic influenza caused by a novel human influenza subtype; it is designed to be read in conjunction with the recommendations (*1*), which are intended as guidance and not formal WHO advice (Appendix) (*2*). The evidence base for recommendations is limited, consisting primarily of historical and contemporary observations, rather than controlled scientific studies. The first part of this series summarized the transmission characteristics of influenza viruses and the basis for interventions to reduce international spread (*3*). This second part addresses measures at the national and community levels that are intended to reduce exposure of susceptible persons to the novel virus. The observations that pandemics do not infect all susceptible persons in the first wave and that subsequent waves occur suggest that preventing disease by reducing exposure is an achievable objective (*3*). By limiting exposure, people who are not infected during the first wave may have an increased chance of receiving virus-specific vaccine as it becomes available. In addition, if the virus becomes less virulent over time, persons who fall ill in subsequent waves may have milder illnesses. This article does not address public communication or infection-control measures for patient care (*4**,**5*).

## Measures To Reduce Spread within Populations

### Isolation of Patients and Quarantine of Contacts

#### Community Level

Reports from many countries indicate that mandatory case reporting and isolating patients during the influenza pandemic of 1918 did not stop virus transmission and were impractical. In Canada, the medical officer of health for the province of Alberta concluded that forced home isolation of patients, posting signs on houses, and "quarantine" (details unspecified) captured only ≈60% of patients in the community because of diagnostic difficulties involving mild cases and failure to notify cases to authorities. As the medical officer noted, "many citizens regarded the placard [sign outside the quarantined person's house] as an injustice, either because they did not believe the diagnosis justified, or because their neighbors were alleged by them to be avoiding quarantine by concealment or evasion… Charges of discrimination were frequently made against the health department" (*6*).

In the Australian state of New South Wales, compulsory reporting was deemed helpful to identify the first introduction of cases into a community. However, once the number of cases grew, reporting cases was not useful or feasible. Also, mild cases were not reported. Compulsory home isolation (which automatically followed reporting) prevented neighbors from bringing needed assistance and was replaced by requesting patients to remain at home (*7*). The reports do not assess the potential impact that requests for ill persons to remain at home voluntarily could have on the reduction of disease within the community.

#### Closed Settings

In closed settings (e.g., military barracks and college dormitories), early identification and isolation of patients in 1918 usually did not completely stop virus transmission but appeared to decrease attack rates, especially when supplemented by restrictions on travel to and from the surrounding community (*8*). In 1 report, 2 sections (A and B) of the student army training corps at the University of Chicago were housed in similar dormitories and fraternity houses, but they had separate classrooms and eating places and no formal contact with each other. In section A, the men received frequent instructions to report illness; all ill persons with "simple colds" or suspected influenza were immediately isolated in hospitals or sent home. In section B, "more or less close contact between sick and well members" was maintained for several days. Lectures and classes were held as usual. From October 17 to November 8, 1918, a total of 26 of 685 men in section A had influenza (attack rate 39/1,000), which was one tenth the attack rate for section B (398/1,000, 93/234 men). New cases ceased in section B after daily inspection and patient isolation were implemented, but these measures were taken late in the epidemic. Among 82 other students living at home or in boarding houses, 7 became ill with influenza (*9*). Similarly, an Australian Quarantine Service review of ship epidemics in 1918 and 1919, including ships quarantined at ports of entry, indicates that daily temperature checks and immediate isolation of patients did not completely prevent transmission but may have reduced the number of cases (*3*).

Reports from several countries (e.g., Australia, Canada, British-occupied Togo) refer to "isolation of contacts" (the preferred modern terminology is isolation of patients and quarantine of contacts) in 1918 and 1919. Details are unclear, but these reports imply that contacts were confined at home. Such measures were consistently described as ineffective and impractical (*6**,**7**,**10*)

Some of the lessons learned from the 2003 severe acute respiratory syndrome (SARS) epidemic can be applied to influenza, including the success of public campaigns to encourage self-recognition of illness, telephone hotlines providing medical advice, and early isolation when potential patients seek health care. Thermally scanning intercity travelers was inefficient in detecting cases. Early isolation of patients and quarantine of contacts successfully interrupted SARS transmission, but influenza's shorter serial interval and earlier peak infectivity, plus the presence of mild cases and possibility of transmission without symptoms, suggest that these measures would be considerably less successful than they were for SARS (*3**,**11**,**12*).

### Social Distancing Measures

#### Avoiding Crowding

A WHO consultation in 1959 concluded that the 1957 influenza pandemic tended to appear first in army units, schools, and other groups where contact was close. Also noting the reduced incidence in rural areas, the consultation suggested that avoiding crowding could reduce the peak incidence of an epidemic and spread it over many, rather than a few, weeks (*13*).

#### Closing Schools and Childcare Centers

A 1959 WHO consultation concluded, "In the Northern hemisphere at least, the opening of schools after the summer holidays seems to have played an important role in initiating the main epidemic phase" (*13*). Despite the propensity of influenza epidemics to be amplified in primary schools (*14*), data on the effectiveness of school closures are limited. Apparently no data or analyses exist for recommending illness thresholds or rates of change that should lead to considering closing or reopening schools.

During a 2-week teachers' strike during an influenza epidemic in Israel in 2000, significant decreases were seen in the rates of diagnoses of respiratory infections, medication purchases, and other parameters for children 6–12 years of age; when school reopened, rates for these parameters rose again (*15*). The study did not report on illness in family members. In 21 regions of France from 1984 to 2000, a temporal relationship was reported between school holidays and a decrease in the incidence of influenza diagnoses by general practitioners 10–20 days later and the daily death rate 30–40 days later, although the time delay raises the question of whether outbreaks may have been subsiding on their own (*16*).

On a small island in the United States in 1920, the single public school was a focal point for the spread of influenza, and a report from that period concluded that "prompt closure of the school would probably not have prevented the epidemic, but might have delayed it" (*17*). School closure might be less effective in some urban areas than in rural areas because urban children can more easily meet elsewhere: in 1918, more influenza cases developed among pupils in a Chicago school after a holiday than when schools were in session (*9*). In Connecticut, the 3 largest cities (Bridgeport, Hartford, and New Haven) kept schools open under "close medical supervision," and their death rates were reportedly lower than those in some Connecticut cities (New London and Waterbury) that closed their schools (*8*).

Universal influenza vaccination of children is controversial, but its use has provided data that help assess the potential effect of reducing transmission by schoolchildren. For example, in 1968–1969, when 86% of its schoolchildren were vaccinated against influenza, the small town of Tecumseh, Michigan, had one third the illness rate of nearby towns where children were not vaccinated (*18*). In Japan, when most schoolchildren were vaccinated against influenza (1962–1987), excess death in the entire population decreased 3- to 4-fold and rose again when the program was discontinued (*19*).

#### Simultaneous Use of Multiple Measures

Influenza and other respiratory viral infections apparently declined in Hong Kong during the 2003 SARS epidemic, as determined on the basis of a review of viral diagnostic laboratory records (*20*). Public health interventions included closing schools, swimming pools, and other public gathering places; cancelling sports events; and disinfecting taxis, buses, and public places. A high percentage of people wore masks in public and washed hands frequently, and in general, much less social mixing occurred.

Reports from the 1918 influenza pandemic indicate that social-distancing measures did not stop or appear to dramatically reduce transmission, but research studies that might assess partial effectiveness are apparently unavailable. For example, in Lomé, British-occupied Togo, case-patients, suspected case-patients, and contacts were isolated; traffic was halted; schools and churches were closed; public meetings were banned. Despite these and other measures, influenza was well established in Lomé by October (*10*). In Edmonton, Canada, isolation and quarantine were instituted; public meetings were banned; schools, churches, colleges, theaters, and other public gathering places were closed; and business hours were restricted without obvious impact on the epidemic (*21**,**22*). In the United States, a comprehensive report on the 1918 pandemic concluded that closing schools, churches, and theaters was not demonstrably effective in urban areas but might be effective in smaller towns and rural districts, where group contacts are less numerous (*8*).

### Measures for Persons Entering or Exiting an Infected Area

In Australia in 1919, political tensions arose among state governments and between states and the national government as individual states sought to protect themselves. Issues included delayed disease reporting by the initially affected state, controls at interstate borders, resistance to quarantine measures, impoundment of the transcontinental train in the state of Western Australia, and conflict between national and state authorities in the Australian federal system (*23*).

Specific details were recorded by the State of New South Wales (NSW) (*24*): "After the first case was diagnosed in Sydney (capital of NSW State) …and determined to have come from (the) adjacent (state of) Victoria, measures were taken by New South Wales at the interstate border to prevent importation of additional cases. These included at first, prohibition of all inbound land traffic, later replaced by quarantine detention camps at which inbound travelers were required to remain at first 7, later 4 days. Also ships from Victoria State were required to anchor in Sydney harbor for 4 days, after which disembarking persons were medically inspected. After Sydney had nevertheless become severely affected, (unspecified) restrictions on traveling out of Sydney were also imposed." The report states that any benefits of land quarantine or interstate or intrastate travel restrictions were "very meager."

In Canada in 1918, one report noted, "Many small towns attempted to isolate themselves with complete quarantines, reminiscent of medieval attempts to stave off plague, in which no one was allowed to enter or leave town. No one was allowed to buy railway tickets to these towns and passengers were barred from disembarking at them. The Canadian Pacific Railway reported 40–45 towns closed in the province of Manitoba during the height of the epidemic; the Canadian Northern line bypassed 15 more. The Alberta Provincial Police guarded roadblocks on major highways in the Province of Alberta in an effort to keep influenza from reaching three prairie municipalities. These measures were nonetheless 'lamentably inefficient in checking the spread of the disease.' Quite simply, isolating individuals and families or quarantining entire communities did not work" (*6**,**21**,**22*).

In the United States, some towns in Colorado and Alaska implemented measures, such as a 5-day quarantine on entering travelers, to exclude infected people. Some towns apparently succeeded in escaping the disease, but others did not (*8**,**25*). In July 1921, an explosive outbreak of influenza occurred on the Pacific island of New Caledonia, a French territory. Authorities implicated a ship that had recently arrived at the capital city of Nouméa from Australia, where normal seasonal (winter) influenza cases were occurring. Illness spread rapidly in Nouméa and the southern portion of the colony, in part because of numerous gatherings in celebration of Bastille Day on July 14. However, authorities successfully prevented spread to the isolated northern third of the island. Travel by land to the north was prohibited, a measure that was facilitated by the lack of major roads to the area. Ships leaving Nouméa for the north were required to remain in quarantine for at least 48 hours before departure, and during that time, temperatures of passengers and crew were monitored (*26*).

Recent modeling studies have supported the use of quarantine measures in the unique circumstances of containing an emerging influenza subtype originating in rural Thailand as a supplement to geographically targeting antiviral drugs to the surrounding population. In 1 model, administering antiviral drugs to 90% of people in a 5-km radius within 2 days after detecting illness in 20 persons was estimated to contain a novel subtype with a basic reproduction number (*R*_0_) of 1.5 (*R*_0_ is the mean number of secondary cases generated by 1 infected person in a fully susceptible population). If prophylaxis were supplemented by closing 90% of schools and 50% of workplaces and reducing movement in and out of the affected area by 80%, the model predicted a 90% probability of containment if *R*_0_ = 1.9 (*27*). These additional measures would help overcome shortcomings in case identification and treatment rates; the epidemic could be contained after <200 cases had been detected. Unsuccessful containment nevertheless delayed widescale spread by >1 month in the model. A second modeling study predicted that if every case-patient stayed at home and 70% of susceptible persons remained in their neighborhoods (but no antivirals were given), disease containment would be 98% if *R*_0_ = 1.4 and 57% if *R*_0_ = 1.7 (*28*). These estimates were based on the population structure and interaction dynamics in Thailand and apply to early detection of cases emerging in a rural area.

### Personal Protection and Hygiene Measures

#### Wearing Masks in Public

Apparently no controlled studies assess the efficacy of mask use in preventing transmission of influenza viruses. During the 1918 influenza pandemic, mask use was common and even required by law in many jurisdictions. Skepticism arose, however; the medical officer of health for Alberta, Canada, noted that cases of disease continued to increase after mask use was mandated, and public confidence in the measure's efficacy gave way to ridicule (*6*).

In Australia, mask-wearing by healthcare workers was thought to be protective, and given evidence of transmission in a closed railway carriage, it was concluded that mask wearing "in closed tramcars, railway carriages, lifts, shops, and other in enclosed places frequented by the public had much to recommend it." However, mask-wearing in the open air, as initially required in Sydney, was later thought to be unnecessary (*24*).

In the United States, persons also wore masks as a protective measure. A report from Tucson, Arizona, noted that early measures included "…isolation of ill people, closure of schools, churches, theatres, etc. The epidemic worsened however. As weeks passed, criticism of the measures was expressed, most vocally by businesses losing money but also by religious and educational institutions. To allow some businesses to reopen, city officials ordered 'masks to be worn in any place where people meet for the transaction of necessary business' … (and later by) all persons appearing in public places. Within a few days, there was virtually universal compliance with mask wearing, but the epidemic was subsiding" (*29*).

During the SARS epidemic in 2003, 76% of Hong Kong residents reported wearing masks in public. As noted above, influenza virus isolation rates decreased, but since multiple measures were implemented, the contribution of mask use, if any, is uncertain (*20*). In case-control studies conducted in Beijing and Hong Kong, wearing masks in public was independently associated with protection from SARS in a multivariate analysis. One study found a dose-response effect (*30*). Methodologic limitations of the studies (e.g., retrospective questionnaire design) limit drawing conclusions (*30**,**31*).

#### Hygiene and Disinfection

Recommendations for "respiratory hygiene/cough etiquette," such as covering one's mouth when coughing and avoiding spitting, have been made more on the basis of plausible effectiveness than controlled studies (*32*). As summarized in part 1 of this article, influenza virus can remain viable on environmental surfaces and is believed transmissible by hands or fomites (*3*). Most, but not all, controlled studies show a protective effect of handwashing in reducing upper respiratory infections (Table A1). Most of the infections studied were likely viral, but only a small percentage were due to influenza (*33*). No studies appear to address influenza specifically. In addition, only 1 study (in Pakistan) has been conducted on the effect of handwashing on severe disease (*34*). Most studies have been in care or institutional settings and involve children; the few involving adults were of college students and military recruits. Antibacterial handwashing products do not offer an advantage over soap and water. In the SARS outbreak in Hong Kong in 2003, a case-control study found that washing hands >10 times per day and "disinfecting living quarters thoroughly" (not otherwise defined and reported retrospectively by telephone) appeared to be protective in a multivariate analysis (*31*).

## Discussion

The knowledge base for use in developing guidance for nonpharmaceutical interventions for influenza is limited and consists primarily of historical and contemporary observations, supplemented by mathematical models, rather than controlled studies evaluating interventions. Accordingly, WHO guidance is subject to revision based on additional information. Aside from transmission characteristics of the pandemic strain, which can be estimated but not completely known before a pandemic is under way, guidance for interventions at the national and community level depends on the phase of the pandemic, the severity of disease (a more virulent strain will justify more socially demanding measures), and the extent of transmission in the particular country and community. Animal sources of virus that has been linked to human infection should be controlled and human exposure to infected animals minimized (*35*). In phases 4 and 5 of the pandemic-alert period, which is characterized by limited and highly localized human-to-human transmission, aggressive measures to detect and isolate case-patients and to quarantine their contacts are recommended and should be accompanied by restrictions on movement in and out of affected communities and consideration of geographically targeted antiviral therapy. These measures, however, are considered much less likely to be feasible in an urban population (*1**,**3**,**27*).

The prediction from mathematical models that an emerging novel human influenza virus subtype might be containable at a point of origin in rural Southeast Asia in phases 4 and 5 through the targeted use of antivirals and application of public health measures was not intended to apply once a pandemic has begun or to address other situations (for example, when a pandemic strain enters into a new country at multiple loci) (*27**,**28*). After increasing and sustained transmission occurs in the general population of even 1 country (phase 6, pandemic period), eventual worldwide spread is considered virtually inevitable, and the public health response focus would shift to reducing impact and delaying spread to allow time for vaccine development and institution of other response measures. Part 1 of this article dealt with measures at the international level, but community-level measures outlined in this part of the article will likely have a greater effect, as was true for SARS in 2003. Over time, the changing conditions during a pandemic will require a change in the public health response and recommended interventions, and the need for such changes will present a difficult but critical communications challenge.

Field studies coordinated by WHO will be needed to assess virus transmission characteristics, amplifying groups (e.g., children vs. adults), and attack and death rates. Information on these factors will be needed urgently at the onset of a pandemic because the pandemic subtype may behave differently than previous pandemic or seasonal strains. Such studies will also be needed throughout the pandemic period to determine if these factors are changing and, if so, to make informed decisions regarding public health response measures, especially those that are more costly or disruptive.

Evidence and experience suggest that in pandemic phase 6 (increased and sustained transmission in the general population), aggressive interventions to isolate patients and quarantine contacts, even if they are the first patients detected in a community, would probably be ineffective, not a good use of limited health resources, and socially disruptive. During phase 6, ill persons should be advised to remain at home, if possible, as soon as symptoms develop (and their caregivers should be advised to take appropriate precautions [5]), but doing so would likely require financial and other support for those off work with illness. Measures to increase social distance should be considered in affected communities, depending on the epidemiology of transmission, severity of disease (case-fatality ratio), and risk groups affected. Nonessential domestic travel to affected areas should be deferred if large areas of a country remain unaffected, but enforcing domestic travel restrictions is considered impractical in most cases.

Handwashing and respiratory hygiene/cough etiquette (*32*) should be routine for all and strongly encouraged in public health messages; such practices should be facilitated by making hand-hygiene facilities available in schools, workplaces, and other settings where amplification of transmission would be expected. WHO has recommended that mask use by the public should be based on risk, including frequency of exposure and closeness of contact with potentially infectious persons; routine mask use in public places should be permitted but not required. This recommendation might be interpreted, for example, as supporting mask use in crowded settings such as public transport. The use of masks or respirators, as well as other precautions, for occupationally exposed workers also depends on risk and is beyond the scope of this review (*4**,**5*). Disinfection of household surfaces likely to be contaminated by infectious secretions appears worthwhile, but no evidence supports the efficacy of widespread disinfection of the environment or air. The legal authority and procedures for implementing interventions should be understood by key personnel before a pandemic begins, and all such measures should respect cultural differences and human rights (*1**,**36*).

The need is urgent for additional research on transmission characteristics of influenza viruses and the effectiveness of nonpharmaceutical public health interventions. Such research should include epidemiologic and virologic studies and field assessments of effectiveness and cost, supplemented by modeling studies and historical inquiry. Such research could be undertaken during epidemics of seasonal influenza, and some research investment now being devoted to influenza should be dedicated to this end. Research needs include evaluating the effectiveness of mask use and cough etiquette and evaluating interventions in terms of cases detected and prevented, cost, and effectiveness in alleviating public concerns. Research is also needed to identify ways to make quarantine and other restrictions more focused and less burdensome for individual persons and societies and to assess how "leaky" restrictions can be and still be effective. Improved methods are also needed to communicate with essential partners and the public. Finally, improved informatics capabilities would allow outbreaks to be monitored and interventions to be assessed in real time to meet the needs of all who will help control future pandemics.

## Appendix

### Examples of Nonpharmaceutical Public Health Interventions

Examples of nonpharmaceutical interventions that may be considered during influenza pandemic phases 4, 5, and 6 (37, derived from [38], which contains additional information and the complete World Health Organization recommendations).

### Phases 4 and 5 (Virus is Becoming More Transmissible among Humans)

Rapid detection and isolation of persons infected with the novel subtype.

Tracing of close contacts during the patient's first two weeks of illness and voluntary quarantine of symptomatic persons for one week.

Use of antiviral drugs for treatment of cases and prophylaxis of others in the affected area.

Restriction on the movement of persons in and out of the affected area.

Screening of travelers departing from areas where clusters of human cases are occurring.

### Phase 6 (Pandemic Declared)

Patient isolation and tracing and quarantine of contacts should cease, as such measures will no longer be feasible or useful.

Persons with fever and respiratory symptoms and their contacts should be asked to undergo voluntary home confinement.

Populations in countries with cases should be asked to defer nonessential domestic travel to affected parts of the country.

Countries should provide incoming travelers with health alert notices describing symptoms and where to report should these symptoms develop.

Countries with cases may introduce exit screening measures for departing travelers. However, such measures are disruptive and costly and will not be fully efficient, as influenza viruses can be carried by asymptomatic persons, who will escape detection during screening.

For persons known to have been exposed in an aircraft or aboard a large cruise ship, consideration can be given to recommended daily fever checks among passengers and crew and prophylactic treatment with antiviral drugs, when available.

"Social distancing" measures, such as the closing of schools or cancellation of large gatherings, may be recommended if evidence indicates an association of certain settings or events with amplified transmission or dispersion into the wider community.

Populations should be repeatedly informed of the need for frequent handwashing with soap and water.

Populations should be repeatedly informed of the need for "respiratory hygiene" (covering mouth when coughing or sneezing, careful disposal of soiled tissues or other materials).

Mask wearing by the general population is not expected to have an appreciable impact on transmission, but should be permitted, as this is likely to occur spontaneously.

## Appendix

**Table A1:** Controlled studies of the effect of handwashing on transmitting respiratory infections

First author	Nature of study	Pertinent results	Pertinent conclusions
Carabin (*39*)	Randomized, controlled trial in 47 daycare centers (for children <5 years of age) in Quebec, Canada. Randomization was by center after stratification by incident rate of respiratory infection. Intervention was increased handwashing in children and staff by a single staff training session. Outcomes were upper respiratory tract infections and diarrheal disease in children (measured coliform contamination but no viral microbiology, winters of 1996 and 1997).	Outcome measures were recorded in intervention and control groups in each center in the autumn of 1996 (before intervention) and 1997 (after intervention). Compliance was measured and showed that the intervention had been carried out. Both groups had a decrease in respiratory infections and diarrheal disease; however, intervention groups experienced greater and significantly reduced rates after intervention than control centers. The reduction in upper respiratory infections was 25%, but little effect on diarrheal illnesses was seen. Environmental contamination (with coliforms) was reduced in both groups during the intervention, which suggests spillover of the intervention.	Handwashing reduced the incidence of upper respiratory infections in children <5 years of age.
Dyer (*40*)	10-week cross-over intervention study among 420 schoolchildren (5–12 years of age) in California compared handwashing and enhanced supervised handwashing and use of a hand sanitizer. Outcome measures were absences due to infectious diseases (no microbiology, early spring 1998).	School absences due to infectious diseases during the enhanced handwashing period were 42% lower than in the ordinary period. For absences due to gastrointestinal disease and respiratory infections, the reductions were 29% and 50%, respectively. The effect was consistent in both periods of the trial, and all reductions were significant.	Enhancing handwashing and use of hand sanitizers among children in school reduces infection.
Falsey (*41*)	US intervention study in 3 eldercare homes with a historical control period. Intervention was to get staff to wash their hands between clients (residents) (virologic studies, winter 1995/1996).	In 3 preintervention years, rates of respiratory infection in the elderly were 14.5, 12.8, and 10.4, respectively, per 100 person-months, and rates declined significantly (to 5.7) in the intervention year. The equivalent rates for staff were 21.0, 13.9, 11.3, and 9.5, respectively, with no significant decline. Virologic testing indicated only 37 influenza isolations among 392 illness episodes during the 4 years. No change in specific viruses could account for the decline in year 4.	Staff handwashing seemed to be associated with reduced incidence of respiratory infection in the elderly but not in staff; however, the use of a historical control period can be misleading.
Larson (*42*)	Randomized, double-blind, controlled trial in 238 families in an American city compared the effect of antibacterial and conventional soaps and other products. Outcomes were self-reported symptoms (no microbiology, 48 weeks in an unstated year).	Most symptoms were respiratory. No significant differences were seen in runny noses, fever, cough, or sore throat between intervention and control families.	No advantage to using antibacterial versus conventional washing materials was found in this industrialized country setting.
Luby (*43*)	Community-cluster, randomized, controlled trial in urban setting (Karachi, Pakistan) compared handwashing promotion in all family members with outcomes of diarrheal disease and lower respiratory tract infections (no microbiology, 12 months, 2002–2003).	Children <15 years of age in intervention clusters had lower incidences of cough and breathing difficulty compared with a control (no intervention) group. Children <5 years of age had lower rates of pneumonia, diarrhea, and impetigo in the intervention versus control groups. No advantage of using antibacterial versus ordinary soap was seen.	In this study in a developing country, handwashing had a significant effect in protecting children against respiratory infections of unknown cause. Although most infections would be viral, only a small proportion might be expected to be due to influenza virus.
Ponka (*44*)	Open-clustered, unrandomized intervention study in daycare centers in urban setting (Helsinki, Finland) involved 60 centers with 228 controls. The intervention involved training in increased handwashing among children and staff plus other hygiene measures, including cleaning surfaces and toys, toileting hygiene, excluding ill persons, and some instruction of parents. Outcomes were effect on absences due to infections (no microbiology, winter and spring 1999–2000).	For children <3 years of age, intervention centers had significantly fewer absences due to all infections and respiratory infections in the intervention period compared with a baseline period. The crude percentage reduction in absences due to upper respiratory infections was 39% and that due to all infections was 32%. No such effect was seen in the control centers, and no effect was seen in children 3–6 years of age in either intervention or control centers.	An effect of a combination of hygiene measures was seen but only in young children, and handwashing was only 1 measure.
Roberts (*45*)	Randomized, controlled trial in 23 of 26 daycare centers in an Australian city involved 11 intervention centers and 12 control centers. Compliance (handwashing and wiping children's noses) was measured (no microbiology, 1996).	A significantly lower number of episodes of illness was seen in children <2 years of age, with no significant effect in older children or all children. Rates of absence were lower in the intervention centers, but the difference was not significant. Where compliance was measured against illness rates, a 17% reduction in colds was seen in younger (<24 months) children with no effect in older children.	The study did not support the hypothesis that infection rates could be reduced by handwashing, although this finding could be due to poor compliance with the intervention.
Ryan (*46*)	Large observational study using a historical control period was undertaken before, during, an after a handwashing intervention among military recruits in the United States. Outcome measures were compliance rates, reported illness, and outpatient and hospitalization rates (limited microbiology, streptococcal cultures; 1996–1998).	A 45% reduction in reported outpatient (primary care) consultations was seen for respiratory infections, with no effect on hospitalization. Those complying with the intervention had a significantly lower rate of reported respiratory infections than those not complying (3.2 vs. 4.7 episodes per recruit).	Although the intervention had statistically significant effects, this finding must be interpreted cautiously because of the use of a historical control period.
Uhari (*47*)	Randomized, controlled trial in daycare centers in Helsinki, Finland, compared handwashing promotion in staff, children (<5 years of age), siblings (outside the nursery), and parents. Outcomes were all infections and absences (no microbiology, 15 months in 1991 and 1992).	A small but significant difference was seen in all infections and symptoms attributable to respiratory infections (rhinitis and cough) in children (lower in the intervention group). Infection rates were also lower in the staff, but the article does not mention respiratory versus other infections. Parents of children in the intervention groups missed less time from work because of less illness among their children, but no difference was seen in parental or sibling illnesses.	In this study in a well-resourced country, handwashing had a significant effect on protecting children against respiratory infections of unknown cause. No measurable benefit was seen in protecting families against background-level infectious disease by intervening with their children in nurseries. That finding does not exclude an effect during an outbreak or pandemic.
White (*48*)	Randomized, controlled trial in 4 university residence halls (430 students total) in the United States compared handwashing promotion based around an alcohol-based hand sanitizer (2 halls) versus no intervention. Hand sanitizers were available in both groups but not promoted in the control group (no microbiology, autumn 2001).	Somewhat greater handwashing and far greater use of hand sanitizers were seen in intervention than control residences. Intervention groups had 20% less illness overall and lower rates of all respiratory symptoms (including sore throats, stuffy noses, fever, cough).	In this small study, handwashing and use of a hand sanitizer seemed to protect against respiratory illnesses. No conclusion could be drawn about the additional value of the sanitizer.
